# The effect of single-cell knockout of Fragile X Messenger Ribonucleoprotein on synaptic structural plasticity

**DOI:** 10.3389/fnsyn.2023.1135479

**Published:** 2023-03-23

**Authors:** Marie Gredell, Ju Lu, Yi Zuo

**Affiliations:** Department of Molecular, Cell and Developmental Biology, University of California, Santa Cruz, Santa Cruz, CA, United States

**Keywords:** Fragile X syndrome (FXS), FMRP, *Fmr1*, dendritic spine, synaptic plasticity, cell-autonomous

## Abstract

Fragile X Syndrome (FXS) is the best-known form of inherited intellectual disability caused by the loss-of-function mutation in a single gene. The *FMR1* gene mutation abolishes the expression of Fragile X Messenger Ribonucleoprotein (FMRP), which regulates the expression of many synaptic proteins. Cortical pyramidal neurons in postmortem FXS patient brains show abnormally high density and immature morphology of dendritic spines; this phenotype is replicated in the *Fmr1* knockout (KO) mouse. While FMRP is well-positioned in the dendrite to regulate synaptic plasticity, intriguing *in vitro* and *in vivo* data show that wild type neurons embedded in a network of *Fmr1* KO neurons or glia exhibit spine abnormalities just as neurons in *Fmr1* global KO mice. This raises the question: does FMRP regulate synaptic morphology and dynamics in a cell-autonomous manner, or do the synaptic phenotypes arise from abnormal pre-synaptic inputs? To address this question, we combined viral and mouse genetic approaches to delete FMRP from a very sparse subset of cortical layer 5 pyramidal neurons (L5 PyrNs) either during early postnatal development or in adulthood. We then followed the structural dynamics of dendritic spines on these *Fmr1* KO neurons by *in vivo* two-photon microscopy. We found that, while L5 PyrNs in adult *Fmr1* global KO mice have abnormally high density of thin spines, single-cell *Fmr1* KO in adulthood does not affect spine density, morphology, or dynamics. On the contrary, neurons with neonatal FMRP deletion have normal spine density but elevated spine formation at 1 month of age, replicating the phenotype in *Fmr1* global KO mice. Interestingly, these neurons exhibit elevated thin spine density, but normal total spine density, by adulthood. Together, our data reveal cell-autonomous FMRP regulation of cortical synaptic dynamics during adolescence, but spine defects in adulthood also implicate non-cell-autonomous factors.

## 1. Introduction

Fragile X syndrome (FXS) is the most common inherited intellectual disorder (Warren and Nelson, 1994), characterized by a variety of physical, behavioral, and cognitive symptoms (Berry-Kravis, 2002; Turk, 2011). It is caused by the expansion of a CGG trinucleotide repeat in the *FMR1* gene on the X chromosome (Verkerk et al., 1991), which silences *FMR1* transcription (Pieretti et al., 1991). The *Fmr1* global knockout (KO) mouse generated over three decades ago (The Dutch-Belgian Fragile X Consortium, 1994) exhibits a variety of neurological and behavioral phenotypes, including audiogenic seizures, hypersensitivity to auditory stimuli, hyperactivity, repetitive behaviors, and memory deficits (Pietropaolo et al., 2011; Kramvis et al., 2013; Li et al., 2020), mimicking symptoms in FXS patients (Wisniewski et al., 1991; Musumeci et al., 1999; Rais et al., 2018).

The Fragile X Messenger Ribonucleoprotein (FMRP), which is encoded by the *Fmr1* gene, is present in dendrites and dendritic spines (Weiler et al., 1997; Antar et al., 2004; Ferrari et al., 2007), postsynaptic sites important for the induction and maintenance of synaptic plasticity. FMRP is involved in regulating almost all aspects of gene expression (Richter and Zhao, 2021), and is particularly critical for the transportation and local translation of mRNAs that regulate dendritic growth, synaptic development, and plasticity (Bassell and Warren, 2008). Indeed, postmortem examination shows a higher density of long and thin dendritic spines on cortical neurons in FXS patients than in healthy people (Rudelli et al., 1985; Hinton et al., 1991; Irwin et al., 2001; Beckel-Mitchener and Greenough, 2004). *Fmr1* global KO mice also display an increased density of dendritic spines, as well as a higher percentage of immature-appearing spines, than wild type controls (Comery et al., 1997; Irwin et al., 2002; Galvez and Greenough, 2005; McKinney et al., 2005; Grossman et al., 2006). In addition to altered morphology and density, *Fmr1* global KO mice have altered structural dynamics (formation and elimination) of cortical spines in an age-, region-, and cell type-specific manner (Cruz-Martín et al., 2010; Pan et al., 2010; Padmashri et al., 2013; Hodges et al., 2017).

Previous studies have shown that *Fmr1* global KO mice have abnormal neuronal activity pattern and synchronization in the neocortex and the hippocampus (Gibson et al., 2008; Hays et al., 2011; Paluszkiewicz et al., 2011; Arbab et al., 2018; Scharkowski et al., 2018; Cheyne et al., 2019). Such functional abnormalities may have profound impacts on the synaptic circuit. It is well-recognized that many intracellular signaling pathways that regulate spine formation and maturation are activity-dependent (Saneyoshi et al., 2010). As spine elimination has been associated with activity-dependent processes such as long-term depression and competition between active and inactive neighboring synapses (Stein and Zito, 2019), these plasticity mechanisms may translate the anomalous neuronal activities into defective structural plasticity of synapses. Such complex interplay between cellular and network-level mechanisms raises an interesting question: is the alteration in FXS spine structure and dynamics the result of cell-autonomous dysregulation, or of abnormal activities in the neuronal network?

To address this question, we combined viral and mouse genetic approaches to eliminate FMRP from a small, sparse subset of cortical neurons in an *Fmr1* conditional knockout (CKO) mouse line (Mientjes et al., 2006), and performed *in vivo* two-photon imaging of dendritic spines over time to compare the spine dynamics between FMRP-null neurons and controls. We found that FMRP deletion during postnatal development, but not in adulthood, leads to altered spine dynamics in cortical pyramidal neurons (PyrNs), which reveals a crucial cell-autonomous function of FMRP in development. In addition, the density and morphology of spines on neurons with neonatal FMRP deletion only partially replicate the phenotypes in *Fmr1* global KO mice in adulthood, suggesting the contribution of factors extrinsic to individual cells.

## 2. Materials and methods

### 2.1. Experimental animals

The *Fmr1* global KO mouse line (JAX #003025) was obtained from Dr. Stephen T. Warren’s lab at Emory University; the *Fmr1* CKO mouse line (Mientjes et al., 2006) was obtained from Dr. David L. Nelson’s lab at Baylor College of Medicine; the *Thy1*-GFP-M (JAX #007788) mouse line was obtained from The Jackson Laboratory (Bar Harbor, ME, USA). All mice have been maintained in the C57BL6/J (JAX #000664) background for many generations. Mice were group-housed with littermates and maintained on a 12 h light/dark cycle. All animal experiments were carried out in accordance with protocols approved by The Institutional Animal Care and Use Committee of University of California, Santa Cruz. Only male mice were used for experiments.

### 2.2. Virus injection and cranial window implantation in adult mice

Virus injection and cranial window implantation in adult mice (6–8 weeks old) were performed as described previously (Lu et al., 2021). Briefly, the mouse was anesthetized with isoflurane in oxygen (4% for induction and 1.5% for maintenance), then placed on the stereotaxic frame. Ophthalmic ointment was applied to the eyes to prevent desiccation and irritation. Carprofen (5 mg/kg bodyweight, intraperitoneal), buprenorphine (0.1 mg/kg, subcutaneous), enrofloxacin (5 mg/kg, subcutaneous), and dexamethasone (2 mg/kg, intramuscular) were administered. The fur on the top of the head was removed with a blade; the exposed scalp was sterilized with betadine followed by 70% alcohol. A midline scalp incision was made, and the periosteum was gently scraped off from the skull. A circular piece of the skull (centered at AP = –1 mm, ML = 1.5 mm) was removed with a trephine (diameter = 2.3 mm, Fine Science Tools, Foster City, CA, USA) driven by a high-speed micro-drill (Foredom K1070, Blackstone Industries, LLC, Bethal, CT, USA). AAV2/1-hSyn-Cre virus (Addgene 105553-AAV1, 2.6 × 10^13^ gc/ml) or AAV2/1-CaMKII0.4-Cre-SV40 virus (2.94 × 10^13^ gc/ml; The Penn Vector Core, University of Pennsylvania, Philadelphia, PA, USA) was diluted 1:5,000 in sterile saline and then mixed in a 1:1 ratio with AAV2/1-CAG-Flex-EGFP (Addgene 51502-AAV1, 2.96 × 10^13^ gc/ml). A total of 100 nl of the virus mixture was injected into the center of the window at a depth of 0.6 mm from the cortical surface at a rate of 20 nl/min using a custom-made injection system based on a single-axis oil hydraulic micromanipulator (MO-10, Narishige, Tokyo, Japan). The imaging port was made by gluing a circular cover glass (#2, diameter = 2.2 mm) underneath a donut-shaped glass (#1, inner diameter = 2 mm, outer diameter = 3 mm; Potomac Photonics, Inc., Baltimore, MD, USA). The imaging port was mounted so that the bottom cover glass fit snugly into the cranial window and the top glass donut rested above the skull. The imaging port was secured with a UV-cured adhesive (Fusion Flo, Prevest DenPro, Jammu, India) onto the skull. After the solidification of the adhesive, the scalp flaps were closed with suture. Following 2 weeks of recovery and virus incubation, the central piece of the scalp was excised, and a custom-made stainless-steel head-bar was secured over the skull with dental cement (Jet Denture Repair, Lang Dental, Wheeling, IL, USA). The mouse received enrofloxacin, buprenorphine, and dexamethasone once per day for two extra days post-surgery and was allowed to recover for an additional week prior to imaging.

### 2.3. *In vivo* imaging of dendritic spines through the cranial window

*In vivo* imaging of dendritic spines through the cranial window was performed on a two-photon microscope (Ultima Investigator, Bruker Co., Middleton, WI, USA) using a 16x/0.8 NA water-immersion objective (Nikon Instruments, Inc., Melville, NY, USA) and an ultrafast two-photon laser (Mai Tai, Spectra-Physics, Santa Clara, CA, USA) operating at 940 nm wavelength. The mouse was anesthetized with a mixture of ketamine (20 mg/ml) and xylazine (2.0 mg/ml) in 0.9% sterile saline administered intraperitoneally (5 ml/kg bodyweight). It was then placed onto a custom-made holding stage, secured by the head-bar. Prior to the first imaging session, images of blood vessels were taken under a dissection microscope as a reference for subsequent relocations. Stacks of two-photon images were taken at 12x zoom with a z-step size of 1 μm. After the first imaging session, low-magnification image stacks (1x and 4x zoom, z-step size = 3 μm) were taken to facilitate relocation.

### 2.4. Virus injection in neonatal mice

Virus injection in neonatal mice was performed as previously described (Chen et al., 2018). Briefly, the postnatal (P) day 1–3 mouse was cryo-anesthetized by placement on ice. AAV2/1-CaMKII0.4-Cre-SV40 (2.94 × 10^13^ gc/ml; The Penn Vector Core, University of Pennsylvania, Philadelphia, PA, USA) was diluted 1:5,000 in sterile saline and then mixed in a 1:1 ratio with AAV2/1-CAG-Flex-EGFP (Addgene 51502-AAV1, 2.96 × 10^13^ gc/ml). A total of 100 nl of the virus mixture was injected at a rate of 40 nl/min into the primary somatosensory cortex (AP = 1.75 mm from lambda, ML = 1.25 mm; depth = 0.35 mm) through the scalp and the skull. A total of 4 weeks of incubation were allowed before imaging and immunohistochemical experiments.

### 2.5. Thin skull preparation for *in vivo* imaging of dendritic spines

The thin skull procedure was performed on young (1 month old) mice as previously described (Xu et al., 2009). Briefly, the mouse was anesthetized with a mixture of ketamine (20 mg/ml) and xylazine (2.0 mg/ml) in 0.9% sterile saline administered intraperitoneally (5 ml/kg body weight). Ophthalmic ointment was applied to the eyes to prevent desiccation and irritation, and the fur over the scalp was removed with a blade. A midline incision was made through the scalp and the periosteum was gently scraped off from the skull. A high-speed micro-drill (Foredom K1070, Blackstone Industries, LLC, Bethal, CT, USA) and a microblade were used to thin a small region of the skull to ∼20 μm thickness. A custom-made head-plate with a central opening was attached to the skull by cyanoacrylate glue (Krazy Glue, Elmer’s Products, Westerville, OH, USA), centered over the thinned region. The head-plate was secured onto a custom-made metal baseplate to stabilize the mouse’s head during imaging. Two-photon imaging was performed as described above. After imaging, the head-plate was detached from the skull, the skull was cleaned with sterile saline, and the scalp was sutured.

### 2.6. Dendritic spine data analysis

Images were analyzed using ImageJ as described previously (Xu et al., 2009). A spine was considered eliminated if it was present in the initial image but not in the subsequent image. A spine was considered to have newly formed if it was not present in the initial image but present in the subsequent image. The percentage of spines eliminated/formed was calculated as the number of spines eliminated/formed over the total spines counted from the first imaging session. Spine density was measured by dividing the number of spines on a dendritic segment by the length of the segment. Spines were classified into four morphological categories (mushroom, stubby, thin, and other) as previously described (Hodges et al., 2017).

### 2.7. Immunohistochemistry

The mouse was transcardially perfused with 4% paraformaldehyde (PFA) in 0.01 M phosphate buffered saline (PBS). The brain was removed and post-fixed in 4% PFA overnight at 4^°^C. For all experiments, the brain was cut into 40 μm sections using a vibratome (VT1000S, Leica Biosystems, Deer Park, IL, USA). Sections were permeabilized and blocked with 0.5% Triton X-100 and 10% normal goat serum in PBS, then incubated with rabbit anti-FMRP (1:1,000; F4055, Sigma-Aldrich, St. Louis, MO, USA) and mouse anti-NeuN (1:1,000; MAB377, MilliporeSigma, Burlington, MA, USA) in 0.5% Triton X-100 in PBS at 4^°^C overnight. Sections were then incubated with goat anti-rabbit secondary antibody conjugated to Alexa Fluor 594 (1:1,000; A11037, Life Technologies, Carlsbad, CA, USA) and goat anti-mouse secondary antibody conjugated to Alexa Fluor 647 (1:1,000; A21235, Life Technologies, Carlsbad, CA, USA) in 10% normal goat serum in PBS for 2 h at room temperature. After rinsing in PBS, sections were incubated in 4’,6-diamidino-2-phenylindole (DAPI, 1:36,000) for 15 min. Sections were then mounted with Fluoromount-G mounting medium (Cat# 0100-01, SouthernBiotech, Birmingham, AL, USA). Images were captured with a Zeiss Axiolmager Z2 widefield fluorescence microscope using a 2.5x/0.12 NA or 10x/0.45 NA, or with a Zeiss 880 confocal microscope using a 20x/0.8 NA air objective. The density of neurons with GFP, FMRP, or NeuN labeling was quantified using Neurolucida Explorer 11 (MBF Bioscience, Williston, VT, USA). Individual cells were analyzed for the presence of GFP, FMRP, and NeuN.

### 2.8. Statistical analysis

Statistical analyses were performed using GraphPad Prism 9.3.1 (GraphPad Software, Boston, MA, USA). The Shapiro–Wilk test was used to test for normality. If samples passed the normality test, Student’s *t*-test was used for two-sample comparison; otherwise Mann-Whitney test was used. For multi-sample comparison, one-way or two-way ANOVA was used, followed by post-hoc Dunnett’s or Šidák test (compared with the control group). The sample difference was considered significant if *p* < 0.05. Data are presented as mean ± s.e.m.

## 3. Results

### 3.1. Virus-induced FMRP knockout in single neurons in adolescent and adult mice

**FIGURE 1:**
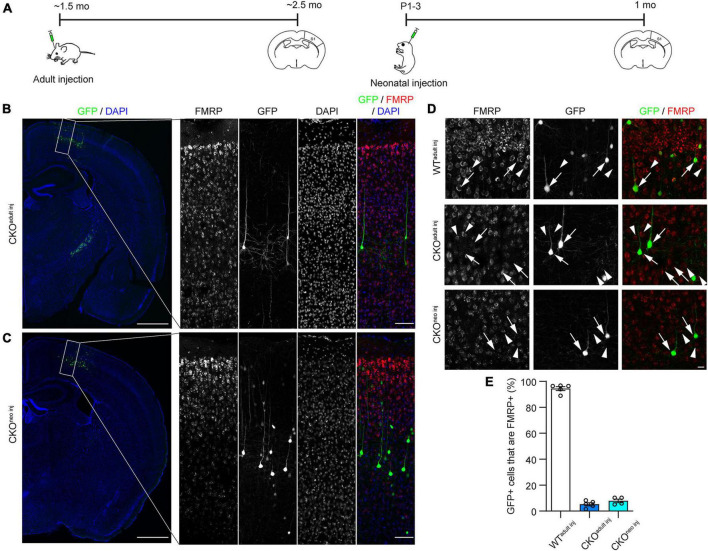
Cre-lox recombination strategy successfully eliminates Fragile X Messenger Ribonucleoprotein (FMRP) from individual infected cells. **(A)** Timeline of virus injection and histology. **(B)** Left: Example of Cre-dependent green fluorescent protein (GFP) expression and *Fmr1* knockout (KO) in a sparse subset of S1 L5 PyrNs of a *Fmr1* conditional knockout (CKO)^adult^ ^inj^ mouse. Scale bar: 500 μm. Right: Enlarged view of the rectangular region in the left panel showing FMRP and GFP expression. Scale bar: 100 μm. **(C)** Examples of *Fmr1* KO in a CKO^neo^ ^inj^ mouse, with the same magnification and arrangement as in **(B)**. **(D)** FMRP and GFP expression in WT^adult^ ^inj^ (top), CKO^adult^ ^inj^ (middle), and CKO^neo^ ^inj^ (bottom) mice imaged with confocal microscopy. Arrows: GFP+ cells; arrowheads: GFP-/FMRP+ cells. Scale bar: 20 μm. **(E)** Percentages of cells co-expressing GFP and FMRP in WT^adult^ ^inj^, CKO^adult^ ^inj^, and CKO^neo^ ^inj^ mice. WT^adult^ ^inj^ *n* = 5 mice (335 cells); CKO^adult^ ^inj^ *n* = 5 mice (397 cells); CKO^neo^ ^inj^ *n* = 4 mice (404 cells).

To investigate whether FMRP regulates the structural plasticity of synapses cell-autonomously, we knocked out FMRP from a sparse subset of layer 5 (L5) PyrNs in the primary somatosensory cortex (S1) of *Fmr1* CKO mice. We chose to target S1 because previous studies have revealed altered tactile information processing (Juczewski et al., 2016; He et al., 2017) and abnormal dendritic spine development (Galvez and Greenough, 2005; Till et al., 2012) in this area of adult *Fmr1* global KO (“GKO”) mice. We accomplished this by injecting a mixture of highly diluted adeno-associated virus (AAV) encoding the Cre recombinase and another AAV encoding floxed green fluorescent protein (GFP) into S1 of CKO mice either in adulthood (∼6 weeks old) or at postnatal day 1-3 (P1-3; Figure 1A). Hereafter we will refer to these mice as “CKO^adult^ ^inj^” and “CKO^neo^ ^inj^,” respectively. This strategy removes the promoter region and the first exon of the *Fmr1* gene via Cre-dependent recombination, thus preventing *Fmr1* transcription. At the same time, the Cre-dependent GFP expression allows us to visualize the cells in which *Fmr1* has been knocked out. We verified the specificity and effectiveness of this strategy with immunohistochemistry (Figures 1B–D). After 3 weeks of virus incubation, we found in CKO^adult^ ^inj^ mice, a sparse subset of L5 PyrNs were GFP+ (Figures 1B, D). Among these cells, only 5.3 ± 1.2% were FMRP+ (Figure 1E). Similarly, CKO^neo^ ^inj^ mice exhibited sparse GFP labeling of L5 PyrNs (Figures 1C, D), and only 7.3 ± 1.1% of such cells were FMRP+ (Figure 1E). In contrast, wild type (WT) mice that received the same virus injection in adulthood (“WT^adult^ ^inj^”) continued to express FMRP in infected cells, with 94.5 ± 1.5% of GFP+ cells being FMRP+ (Figure 1E). These data confirm the effectiveness and specificity of our knockout strategy.

### 3.2. Single-cell FMRP knockout does not alter spine density

**FIGURE 2:**
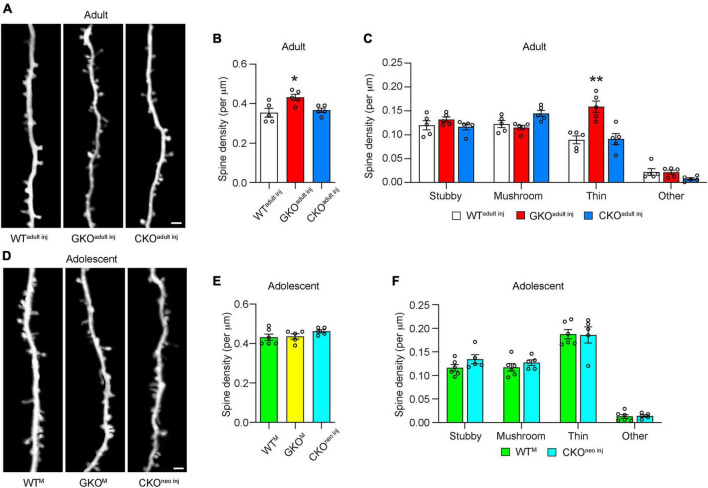
Cell-autonomous *Fmr1* knockout (KO) in adolescence or adulthood does not alter spine density. **(A)** Examples of dendritic spines imaged *in vivo* in adult mice. **(B)** Total spine density in WT^adult^ ^inj^, global knockout (GKO*^adultinj^*), and conditional knockout (CKO*^adultinj^*) mice. *n* = 5 per group. **(C)** Density of different types of spines in WT^adult^ ^inj^, GKO^adult^ ^inj^, and CKO^adult^ ^inj^ mice. *n* = 5 per group. **(D)** Examples of dendritic spines imaged *in vivo* in adolescent mice. **(E)** Total spine density in adolescent mice. *n* = 6 for WT^M^, 5 for GKO^M^, and CKO^neo^ ^inj^ mice. **(F)** Density of different types of spines in adolescent mice. *n* = 6 for WT^M^ and 5 for CKO^neo^ ^inj^ mice. Scale bar = 2 μm. Hereinafter *p < 0.05, **p < 0.01; post-hoc comparisons with the control group.

To assess the effects of single-cell *Fmr1* KO on the structural dynamics of dendritic spines, we performed longitudinal *in vivo* two-photon imaging either through a cranial window (Holtmaat et al., 2009) or with the thin-skull preparation (Xu et al., 2009). We first compared the density of spines on apical dendritic tufts of L5 PyrNs in WT^adult^ ^inj^, CKO^adult^ ^inj^, and GKO mice receiving virus injection in adulthood (“GKO^adult^ ^inj^”) when the mice reached 10 weeks of age (Figure 2A). We found that in GKO^adult^ ^inj^ mice, the spine density was 0.43 ± 0.02 per μm, significantly higher than that in WT^adult^ ^inj^ mice [0.35 ± 0.02 per μm; one-way ANOVA, *F*(2,12) = 6.111, *p* < 0.05*;* post-hoc Dunnett’s multiple comparisons test *p* < 0.05; Figure 2B], which is consistent with reports in the literature (Galvez and Greenough, 2005). We further analyzed the density of spines in different morphological categories (Figure 2C). Only thin spines exhibited significant density difference between GKO^adult inj^ and WT^adult inj^ mice [two-way repeated measures ANOVA, *F*(6, 36) = 10.02, *p* < 0.0001; post-hoc Dunnett’s multiple comparisons test *p* < 0.01]; the rest showed no difference (post-hoc Dunnett’s multiple comparisons test *p* = 0.4707, 0.6510, and 0.9983 for stubby, mushroom, and others, respectively). Interestingly, spine density in CKO^adult^ ^inj^ mice (0.37 ± 0.01 per μm) was not significantly different from that in WT^adult^ ^inj^ mice (post-hoc Dunnett’s multiple comparisons test *p* = 0.8378; Figure 2B). Nor was there significant density difference in spines belonging to any morphological category between CKO^adult inj^ and WT^adult inj^ mice (post-hoc Dunnett’s multiple comparisons test *p* = 0.9518, 0.0955, 0.9908, and 0.1754 for stubby, mushroom, thin, and others, respectively). To control for the possibility that virus infection *per se* affects spine density, we also measured spine density in *Thy1*-GFP-M mice (which are *Fmr1*+, hence denoted “WT^M^”) as well as in GKO × *Thy1*-GFP-M (“GKO^M^”) mice. These animals express cytoplasmic GFP in a sparse subset of cortical L5 PyrNs, thus obviating the need for viral labeling. We found no difference in spine density between WT^adult^ ^inj^ mice and WT^M^ mice, or between GKO^adult^ ^inj^ and GKO^M^ mice (Supplementary Figure 1). These results demonstrate that the viral knockout strategy *per se* does not affect spine density.

We next compared the spine density of adolescent (∼P30) WT^M^, GKO^M^, and CKO^neo^ ^inj^ mice (Figure 2D). We found no significant difference among these three groups: the spine density was 0.43 ± 0.02 per μm in WT^M^, 0.44 ± 0.01 per μm in GKO^M^, and 0.46 ± 0.01 per μm in CKO^neo^ ^inj^ mice [one-way ANOVA, *F*(2,13) = 1.421, *p* = 0.2766; Figure 2E]. This agrees with previous findings (Nimchinsky et al., 2001; Galvez and Greenough, 2005; Pan et al., 2010; Hodges et al., 2017; Bland et al., 2021). Furthermore, CKO^neo inj^ mice showed no significant difference in the density of any spine type in comparison with WT^M^ mice (two-way repeated measures ANOVA, *F*(3,27) = 0.4985, *p* = 0.6864; Figure 2F), similar to the previous report on adolescent GKO mice (Hodges et al., 2017).

### 3.3. Neither global nor single-cell knockout of FMRP affects the structural dynamics of dendritic spines in adult mice

**FIGURE 3:**
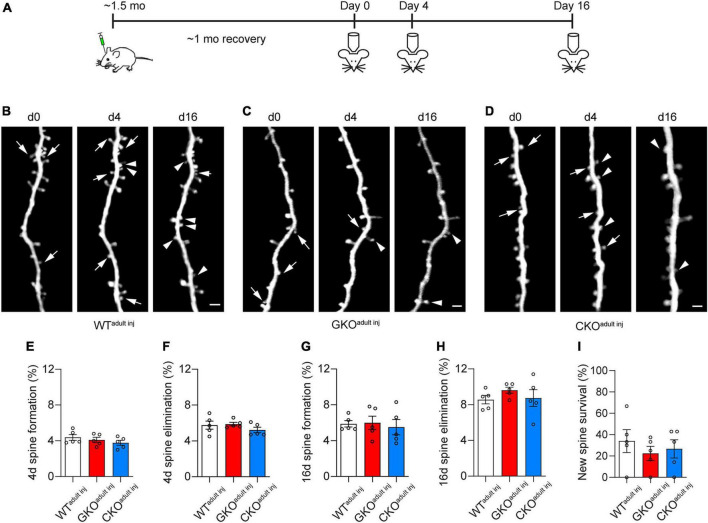
Cell-autonomous *Fmr1* knockout (KO) in adulthood does not affect spine formation or elimination. **(A)** Timeline of virus injection and *in vivo* two-photon imaging. **(B–D)** Examples of spine formation and elimination in WT^adult^ ^inj^ **(B)**, global knockout (GKO^adult^ ^inj^) **(C)**, and conditional knockout (CKO^adult^ ^inj^) **(D)** mice. Arrows: eliminated spines; arrowheads: formed spines. Scale bar = 2 μm. **(E,F)** Spine formation **(E)** and elimination **(F)** rates over 4 days in WT^adult^ ^inj^, GKO^adult^ ^inj^, and CKO^adult^ ^inj^ mice. **(G,H)** Spine formation **(G)** and elimination **(H)** rates over 16 days in WT^adult^ ^inj^, GKO^adult^ ^inj^, and CKO^adult^ ^inj^ mice. **(I)** Percentage of new spines formed by day 4 that survived till day 16. *n* = 5 mice per group.

We then examined the structural dynamics of spines in WT^adult^ ^inj^, GKO^adult^ ^inj^, and CKO^adult^ ^inj^ mice starting at about 2 months of age, over 4 and 16 days intervals (Figures 3A–D). We found no significant difference in the rate of spine formation and elimination over 4 days: the spine formation rate was 4.4 ± 0.3% in WT^adult^ ^inj^, 4.1 ± 0.3% in GKO^adult^ ^inj^, and 3.8 ± 0.3% in CKO^adult^ ^inj^ [one-way ANOVA, *F*(2,12) = 1.017, *p* = 0.3907; Figure 3E], and the spine elimination rate was 5.8 ± 0.4% in WT^adult^ ^inj^, 5.9 ± 0.2% in GKO^adult^ ^inj^, and 5.2 ± 0.3% in CKO^adult^ ^inj^ [one-way ANOVA, *F*(2,12) = 1.122, *p* = 0.3574; Figure 3F]. Likewise, there was no significant difference in spine dynamics over 16 days. The spine formation rate was 5.9 ± 0.4%, 6.0 ± 0.8%, and 5.5 ± 0.9% in WT^adult^ ^inj^, GKO^adult^ ^inj^, and CKO^adult^ ^inj^, respectively [one-way ANOVA, *F*(2,12) = 0.1352, *p* = 0.8748; Figure 3G], and the corresponding spine elimination rate was 8.6 ± 0.5% in WT^adult^ ^inj^, 9.6 ± 0.3% in GKO^adult^ ^inj^, and 8.7 ± 0.9% in CKO^adult^ ^inj^ [one-way ANOVA, *F*(2,12) = 0.7999, *p* = 0.4719; Figure 3H]. Following new spines formed by day 4 till day 16, we found no significant difference in their survival rate [WT^adult inj^ 34.0 ± 10.7%, GKO^adult inj^ 22.5 ± 6.7%, CKO^adult inj^ 26.7 ± 8.5%; one-way ANOVA, *F*(2,12) = 0.4419, *p* = 0.6529; Figure 3I]. These results suggest that deleting FMRP from single neurons in adulthood does not affect its spine dynamics. Again, to control for potential confounding effects of virus infection, we measured spine dynamics in WT^M^ and GKO^M^ mice at comparable ages. Spine dynamics did not differ significantly between WT^adult^ ^inj^ and WT^M^ mice, or between GKO^adult^ ^inj^ and GKO^M^ mice (Supplementary Figure 2). This confirms that the viral labeling strategy does not affect spine dynamics either.

### 3.4. Single-cell FMRP knockout results in elevated dendritic spine formation in adolescent mice

**FIGURE 4:**
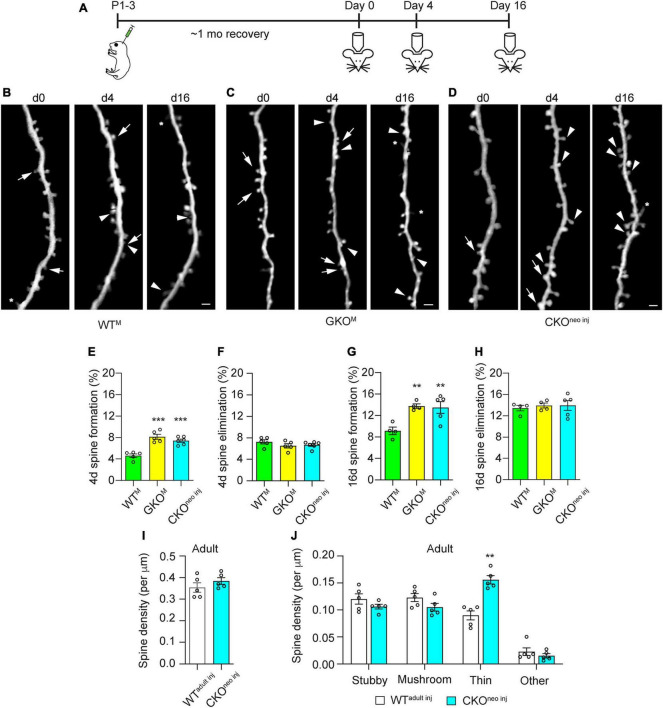
Cell-autonomous *Fmr1* knockout (KO) in adolescence selectively affects spine formation but not elimination. **(A)** Timeline of virus injection and *in vivo* two-photon imaging. **(B–D)** Examples of spine formation and elimination in adolescent WT^M^ **(B)**, global knockout (GKO^M^) **(C)**, and conditional knockout (CKO^neo^ ^inj^) **(D)** mice. Arrows: eliminated spines; arrowheads: formed spines; asterisks: filopodia. Scale bar = 2 μm. **(E,F)** Spine formation **(E)** and elimination **(F)** rates over 4 days in WT^M^, GKO^M^, and CKO^neo^ ^inj^ mice. *n* = 5 mice for WT^M^ and GKO^M^, and six mice for CKO^neo^ ^inj^. **(G,H)** Spine formation **(G)** and elimination **(H)** rates over 16 days in WT^M^, GKO^M^, and CKO^neo^ ^inj^ mice. *n* = 5 mice per group. **(I)** Total spine density in WT^adult^ ^inj^ and CKO^neo^ ^inj^ mice at adulthood. *n* = 5 mice per group. **(J)** Density of different types of spines in WT^adult^ ^inj^ and CKO^neo^ ^inj^ mice at adulthood. *n* = 5 mice per group. **p < 0.01, ***p < 0.001.

As previous studies suggest that *Fmr1* KO affects spine dynamics most prominently in adolescence (Hodges et al., 2017), we examined 4 and 16 days spine dynamics in CKO^neo^ ^inj^, WT^M^, and GKO^M^ mice starting at 1 month of age (Figures 4A–D). We found that spine formation over 4 days was significantly elevated in CKO^neo^ ^inj^ mice (7.4 ± 0.3%) and GKO^M^ mice (8.1 ± 0.5%) compared to WT^M^ mice [4.6 ± 0.3%; one-way ANOVA, *F*(2,13) = 23.71, *p* < 0.001; post-hoc Dunnett’s multiple comparisons test: *p* < 0.001 for CKO^neo^ ^inj^ vs. WT^M^ and for GKO^M^ vs WT^M^; Figure 4E]. However, spine elimination over 4 days was unaffected [WT^M^ 7.2 ± 0.4%, GKO^M^: 6.5 ± 0.4%, CKO^neo inj^: 6.7 ± 0.3%; one-way ANOVA, *F*(2,13) = 1.225, *p* = 0.3255; Figure 4F]. A similar phenomenon emerged over the 16 days interval: CKO^neo^ ^inj^ mice had a spine formation rate of 13.5 ± 1.1%, which was comparable to that in GKO^M^ mice (13.7 ± 0.3%) but differed significantly from that in WT^M^ mice [9.2 ± 0.5%; one-way ANOVA, *F*(2,12) = 12.05, *p* < 0.01; post-hoc Dunnett’s multiple comparisons test: *p* < 0.01 for both CKO^neo^ ^inj^ vs WT^M^ and GKO^M^ vs. WT^M^; Figure 4G]. The 16 days spine elimination did not significantly differ among the three groups, with rates of 13.3 ± 0.4% (WT^M^), 13.8 ± 0.4% (GKO^M^), and 13.9 ± 0.9% (CKO^neo inj^), respectively [one-way ANOVA, *F*(2,12) = 0.3124, *p* = 0.7374; Figure 4H].

We further followed CKO^neo inj^ mice into adulthood and re-examined their spine density and morphology. To our surprise, spine density on the FMRP-null neurons was comparable to that in WT^adult inj^ mice [unpaired *t*-test, *t*(8) = 1.116, *p* = 0.2966; Figure 4I]. Morphological analysis, however, revealed an elevated density of thin spines on FMRP-null neurons [two-way repeated measures ANOVA, *F*(3,24) = 22.56, *p* < 0.0001; post-hoc Šidák multiple comparisons test, *p* < 0.01; Figure 4J]. The normalization of total spine density was due to decreased density of all other types of spines. Together with findings in adult animals, these results suggest that FMRP regulates the structural dynamics of spines cell-autonomously in adolescence, but the development of spine defects into adulthood also involves non-cell-autonomous factors.

## 4. Discussion

The abundance of dendritic spines with an immature morphology in the adult brain is an anatomical hallmark of FXS in humans and in mouse models. Traditionally, it is conjectured that this phenotype results from defective spine pruning during development (Greenough et al., 2001; Churchill et al., 2002; Bagni and Greenough, 2005; Bardoni et al., 2006). More recent *in vivo* imaging studies, however, reveal elevated spine formation in adolescence (Pan et al., 2010; Padmashri et al., 2013; Nagaoka et al., 2016; Hodges et al., 2017), and some of them in addition suggest that spine elimination during this developmental stage is elevated as well. The cellular underpinning of such altered structural dynamics is likely complex. It may involve abnormal neural activity patterns operating through activity-dependent mechanisms to prevent the maturation of new spines and the competitive removal of weak and immature spines. It may also implicate altered intrinsic excitability of neurons due to ion channel dysregulation, excitation/inhibition imbalance induced by dysfunctional local inhibitory circuits, and altered homeostatic plasticity (Gibson et al., 2008; Soden and Chen, 2010; Goel et al., 2018; Liu et al., 2021). Moreover, astrocytes may contribute to the spine pathology, as astrocyte-specific KO of *Fmr1* suffices to elevate spine formation (Hodges et al., 2017). Other studies in addition suggests altered inflammatory response of microglia (Parrott et al., 2021) and reduced microglia-mediated synaptic pruning (Jawaid et al., 2018) in *Fmr1* global KO mice. Such a plethora of participants makes it difficult to isolate the contribution of cell-autonomous dysregulation from that of external factors, if *Fmr1* is knocked out globally. In fact, even studies that leverage the random X-linked inactivation of *Fmr1* in heterozygous females to generate mosaicism (approximately half of the neurons are FMRP-null and the other half FMRP+) still suffer from the caveat that network effects cannot be ruled out (Bland et al., 2021).

In this study, we circumvented this problem by a virus-based strategy to induce *Fmr1* KO only in a very small subset of cortical PyrNs. Thus, the perturbation to the activity pattern in the neuronal network is negligible. Furthermore, as each PyrN receives thousands of inputs (Iascone et al., 2020), the vast majority of them are from neurons that express FMRP normally. We observed that in adolescent CKO^neo^ ^inj^ mice, FMRP-null neurons exhibited the same spine dynamics as in *Fmr1* global KO mice, indicating that FMRP regulates spine dynamics cell-autonomously at this developmental stage. This result is consistent with a recent electrophysiological study (Zhang et al., 2021) showing that virus-based cell-autonomous deletion of FMRP from L2/3 or L5 neurons weakens callosal excitatory synapses. It is also consistent with the earlier study (Pfeiffer and Huber, 2007) showing that acute, postsynaptic expression of FMRP in *Fmr1* KO neurons *in vitro* reduces their synapse number. This suggests that in *Fmr1* global KO mice, which more realistically reflect the condition in FXS patients, the lack of FMRP in the neurons to which the spines belong is the determining factor of the pathology in spine density and dynamics. Interestingly, although neurons with neonatal FMRP deletion exhibit abnormally high density of thin spines when the animal reaches adulthood, the total spine density remains at the WT level. In contrast, the elevated density of thin spines on neurons in adult GKO mice increases total spine density as well. This intriguing phenomenon calls for further investigations into the contribution of the neuronal network and other extrinsic factors. It is worth noting that the regulation of spine dynamics by FMRP does not imply that the effect is mediated completely intracellularly. It has been reported that genetic deletion of matrix metalloproteinase-9 (MMP-9), an FMRP target enzyme involved in the degradation of the extracellular matrix, can rescue spine morphological and behavioral deficits in *Fmr1* global KO mice (Sidhu et al., 2014). Another work shows that injecting an MMP-9 inhibitor likewise rescues the baseline spine dynamics in such animals (Nagaoka et al., 2016).

Most *in vivo* imaging studies of spine dynamics in FXS focus on the apical dendrites of L5 PyrNs, leveraging the sparse but very bright neuronal labeling conveniently offered by the *Thy1*-YFP-H or *Thy1*-GFP-M line (Pan et al., 2010; Padmashri et al., 2013; Nagaoka et al., 2016; Hodges et al., 2017). However, there is evidence that FMRP regulates spine morphology differentially in different compartments of the dendritic arbor. For example, a histological study (Bland et al., 2021) shows that *Fmr1* KO or inactivation affects the density of spines on basal dendrites of L5 PyrNs minimally. It will be interesting to examine whether the dynamics of such spines are altered by the loss of FMRP; such experiments have become possible with recent advances in imaging techniques such as three-photon microscopy and adaptive optics (Rodriguez and Ji, 2018; Sinefeld et al., 2022).

The regulatory role of postsynaptic FMRP may also be input-specific. A recent immunofluorescent array tomography study of cortical tissues from adult *Fmr1* global KO mice (Simhal et al., 2019) revealed an increase of small synapses that expressed vesicular glutamate transporter 1 (VGluT1+) in L4 and a decrease of large VGluT1+ synapses in L1 and L4; moreover, VGluT2+ synapse density consistently decreased in L1 and L2/3. As VGluT1+ and VGluT2+ excitatory synapses are generally considered to be corticocortical and thalamocortical, respectively, this work suggests an input-specific defect associated with *Fmr1* KO. More interestingly, it was recently found (Zhang et al., 2021) that, while barrel cortex L2/3 neurons with cell-autonomous *Fmr1* KO had weaker long-range callosal synaptic connections, their excitatory postsynaptic currents (EPSCs) evoked by local inputs (L4 of home or adjacent barrels, L5A or L2/3 neurons) were unaffected. Similarly, L5 PyrNs with postsynaptic *Fmr1* KO had weakened callosal inputs around their somata and apical dendrites. These findings are intriguing, as only a very small percentage of the neurons were FMRP-null, and hence their presynaptic partners should be predominantly normal no matter where they resided. The mechanisms through which postsynaptic FMRP differentially regulate the maturation and strength of synapses from different input sources remain to be elucidated.

## Data availability statement

The raw data supporting the conclusions of this article will be made available by the authors, without undue reservation.

## Ethics statement

This animal study was reviewed and approved by Institutional Animal Care and Use Committee (IACUC), University of California, Santa Cruz.

## Author contributions

YZ, JL, and MG designed the study and wrote the manuscript. MG and JL performed the experiments and analyzed the data. All authors approved the submitted version of the manuscript.
