# Supplementary material for: The effect of single-cell knockout of Fragile X Messenger Ribonucleoprotein on synaptic structural plasticity
Source: Front Synaptic Neurosci. 2023 Mar 23;15:1135479. doi: 10.3389/fnsyn.2023.1135479 (PMC10076639; doi:10.3389/fnsyn.2023.1135479)
Supplement: Supplementary file 1 [file Data_Sheet_1.pdf]

Supplementary Material

The Effect of Single-cell Knockout of Fragile X Messenger Ribonucleoprotein on Synaptic Structural Plasticity

Marie Gredell, Ju Lu, Yi Zuo*

*** Correspondence:** Yi Zuo: yizuo@ucsc.edu

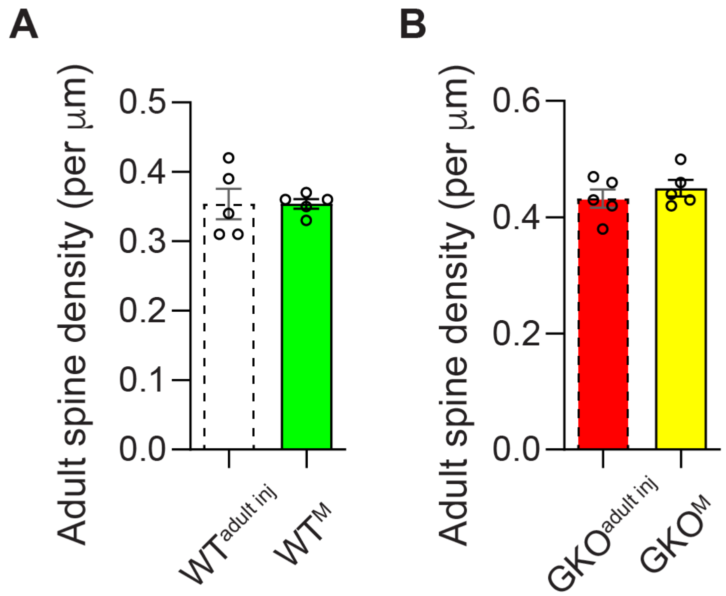

Supplementary Figure 1. Virus injection *per se* does not affect spine density.
A. Spine density does not differ significantly between $WT^{adult\ inj}$ (0.35 ± 0.02 per μm) and WT^{M} (0.35 ± 0.01 per μm) mice. Unpaired *t*-test, $t(8) = 0.000$, $p > 0.999$. **B.** Spine density does not differ significantly $GKO^{adult\ inj}$ (0.43 ± 0.02 per μm) and GKO^{M} (0.45 ± 0.01 per μm) mice. Unpaired *t*-test, $t(8) = 0.8448$, $p = 0.4228$. $n = 5$ mice per group.

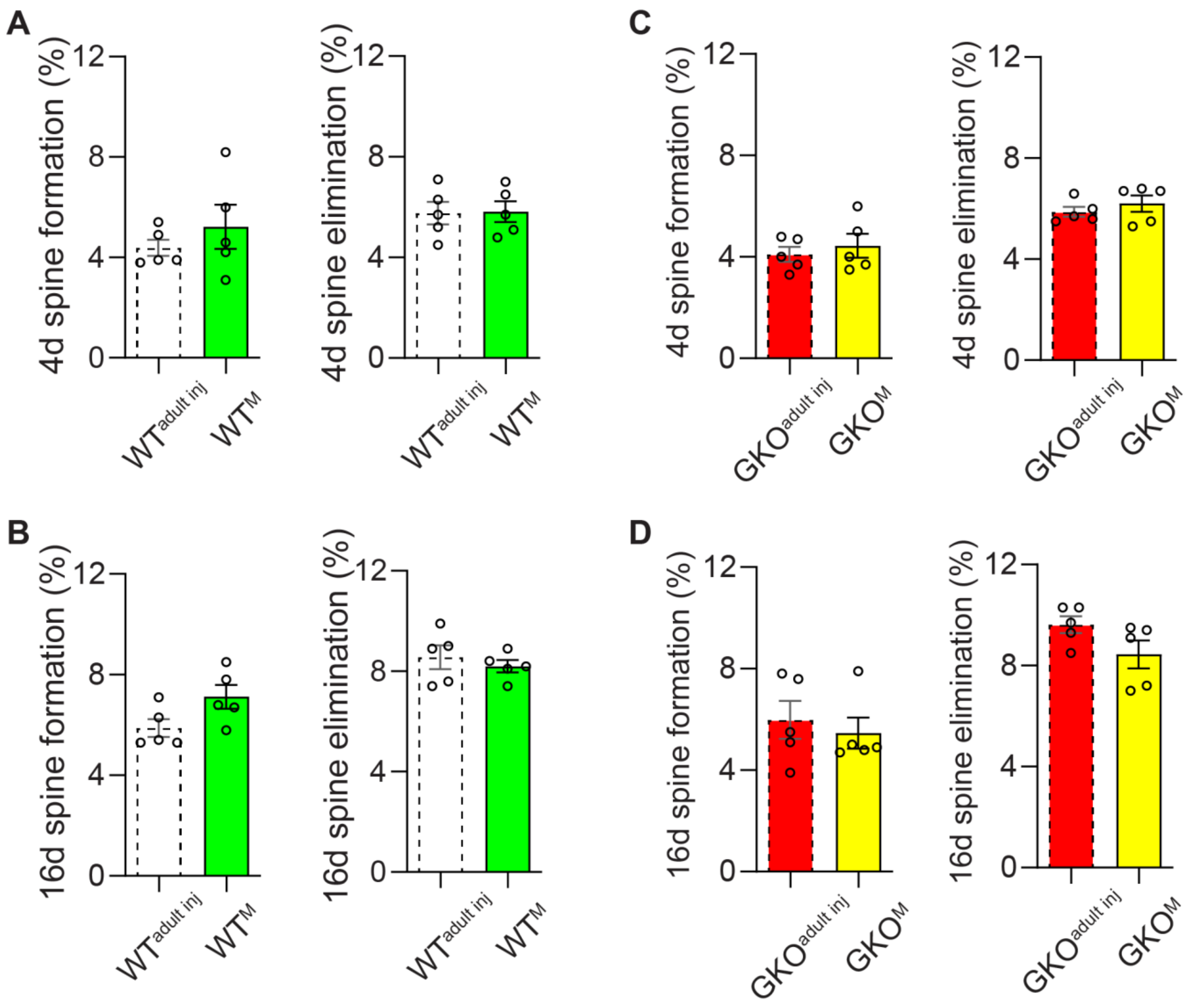

Supplementary Figure 2. Virus injection *per se* does not affect spine dynamics.

A. Spine dynamics over 4 days do not differ between $WT^{adult\ inj}$ and WT^{M} mice. Formation: 4.4 ± 0.3% ($WT^{adult\ inj}$) vs 5.2 ± 0.9% (WT^{M}), unpaired *t*-test, $t(8) = 0.8976$, $p = 0.3956$. Elimination: 5.8 ± 0.4% ($WT^{adult\ inj}$) vs 5.8 ± 0.4% (WT^{M}), unpaired *t*-test, $t(8) = 0.0985$, $p = 0.9240$. **B.** Spine dynamics over 16 days do not differ between $WT^{adult\ inj}$ and WT^{M} mice. Formation: 5.9 ± 0.4% ($WT^{adult\ inj}$) vs 7.1 ± 0.5% (WT^{M}), unpaired *t*-test, $t(8) = 2.103$, $p = 0.0687$. Elimination: 8.6 ± 0.5% ($WT^{adult\ inj}$) vs 8.2 ± 0.2% (WT^{M}), unpaired *t*-test, $t(8) = 0.6833$, $p = 0.5137$. **C.** Spine dynamics over 4 days do not differ between $GKO^{adult\ inj}$ and GKO^{M} mice. Formation: 4.1 ± 0.3% ($GKO^{adult\ inj}$) vs 4.4 ± 0.5% (GKO^{M}), unpaired *t*-test, $t(8) = 0.6191$, $p = 0.5531$. Elimination: 5.9 ± 0.2% ($GKO^{adult\ inj}$) vs 6.2 ± 0.3% (GKO^{M}), unpaired *t*-test, $t(8) = 0.8335$, $p = 0.4287$. **D.** Spine dynamics over 16 days do not differ between $GKO^{adult\ inj}$ and GKO^{M} mice. Formation: 6.0 ± 0.8% ($GKO^{adult\ inj}$) vs 5.5 ± 0.6% (GKO^{M}), Mann-Whitney test, $U = 9$, $p = 0.5476$. Elimination: 9.6 ± 0.3% ($GKO^{adult\ inj}$) vs 8.4 ± 0.6% (GKO^{M}), unpaired *t*-test, $t(8) = 1.823$, $p = 0.1058$. $n = 5$ mice per group.
